# Social Media Posts About Medical Tests With Potential for Overdiagnosis

**DOI:** 10.1001/jamanetworkopen.2024.61940

**Published:** 2025-02-26

**Authors:** Brooke Nickel, Ray Moynihan, Emma Grundtvig Gram, Tessa Copp, Melody Taba, Patti Shih, Raffael Heiss, Mingyao Gao, Joshua R. Zadro

**Affiliations:** 1 Sydney Health Literacy Lab, Sydney School of Public Health, Faculty of Medicine and Health, The University of Sydney, Sydney, New South Wales, Australia; 2 Wiser Healthcare, Sydney School of Public Health, Faculty of Medicine and Health, The University of Sydney, Sydney, New South Wales, Australia; 3 Institute for Evidence-Based Healthcare, Faculty of Health Sciences and Medicine, Bond University, Robina, Queensland, Australia; 4 Center for General Practice, Department of Public Health, University of Copenhagen, Copenhagen, Denmark; 5 Australian Centre for Health Engagement Evidence & Values, School of Health and Society, University of Wollongong, Wollongong, New South Wales, Australia; 6 Center for Social & Health Innovation, Management Centre Innsbruk–The Entrepreneurial School, Innsbruk, Austria; 7 Sydney Musculoskeletal Health and Institute for Musculoskeletal Health, Sydney School of Public Health, Faculty of Medicine and Health, The University of Sydney and Sydney Local Health District, Sydney, New South Wales, Australia

## Abstract

**Importance:**

Social media is an influential source of medical information, but little is known about how posts discuss medical tests that carry potential for overdiagnosis or overuse.

**Objective:**

To investigate how social media posts discuss 5 popular medical tests: full-body magnetic resonance imaging, the multicancer early detection test, and tests for antimullerian hormone, gut microbiome, and testosterone.

**Design, Setting, and Participants:**

This cross-sectional study assessed posts on Instagram and TikTok between April 30, 2015, and January 23, 2024, that discussed full-body magnetic resonance imaging, the multicancer early detection test, and tests for antimullerian hormone, gut microbiome, and testosterone. Using keywords on newly created accounts, posts were searched and screened until 100 posts for each test on each platform were identified (n = 1000). Posts were excluded if they did not discuss 1 of the 5 tests or were not in English or if the account holders had fewer than 1000 followers.

**Main Outcomes and Measures:**

The main outcome was information about benefits, harms, and overall tone discussed in the post. All outcomes were summarized descriptively. Logistic regression was used to assess whether the use of evidence or the account holder being a physician or having financial interests influenced how tests were discussed.

**Results:**

A total of 982 posts from account holders with a combined 194 200 000 followers were analyzed. Across all tests, benefits were mentioned in 855 posts (87.1%) and harms in 144 (14.7%), with 60 (6.1%) mentioning overdiagnosis or overuse. Overall, 823 posts (83.8%) had a promotional (vs neutral or negative) tone. Evidence was explicitly used in 63 posts (6.4%), personal anecdotes were used in 333 (33.9%), 498 posts (50.7%) encouraged viewers to take action and get the test, and 668 account holders (68.0%) had financial interests. Posts from physicians were more likely to mention harms (odds ratio, 4.49; 95% CI, 2.85-7.06) and less likely to have an overall promotional tone (odds ratio, 0.53; 95% CI, 0.35-0.80).

**Conclusions and Relevance:**

In this cross-sectional study of social media posts about 5 popular medical tests, most posts were misleading or failed to mention important harms, including overdiagnosis or overuse. These data demonstrate a need for stronger regulation of misleading medical information on social media.

## Key Points

**Question:**

How do social media posts discuss popular medical tests with potential for overdiagnosis or overuse?

**Findings:**

In this cross-sectional analysis of 982 social media posts from account holders with more than 194 million total followers, 87.1% mentioned benefits, 14.7% mentioned potential harms, 6.1% mentioned overdiagnosis, and 6.4% included scientific evidence. Posts from physicians were more likely to mention harms and less likely to promote the test.

**Meaning:**

These findings suggest that social media posts on medical tests with potential for overdiagnosis or overuse may be overwhelmingly misleading and fail to mention potential harms, indicating an urgent need for effective regulation to protect the public.

## Teaser

This cross-sectional study examines the tone and content of social media posts that discuss popular medical tests with potential for overdiagnosis or overuse.

## Introduction

Systematic review evidence suggests medical misinformation is prevalent across social media platforms and that influencers can impact the health behaviors of their followers.^1,2^ Social media is known to promulgate misleading information about unhealthy products, such as alcohol and junk food, as well as misinformation about medical products.^3,4,5,6^ In 2023, celebrity influencer Kim Kardashian urged her more than 360 million followers to undergo “lifesaving” full-body magnetic resonance imaging (MRI)^7,8^ despite no evidence of benefit for people at average risk of disease and the clear danger of overdiagnosis and overuse.^9,10,11^ As part of broader concerns about the effects of social media, there are increasing calls for more research to better understand medical misinformation on social media in order to more effectively address it.^12,13^ At the same time, regulatory agencies, such as the US Food and Drug Administration, identify inappropriate testing as one cause of unnecessary treatments, which can adversely impact public health, and are moving to better regulate medical tests.^14^ To our knowledge, there is limited research aimed at understanding promotion on social media of medical screening and diagnostic tests, such as full-body MRIs, which carry the potential for unnecessary diagnoses and treatments.

Overdiagnosis and overuse are now recognized as major threats to human health, causing harm and waste and diverting resources from tackling underdiagnosis and undertreatment.^15,16,17^ Overdiagnosis happens when generally healthy people are diagnosed or labeled with a condition or disease that would never cause them harm.^18,19^ Evidence is increasingly revealing that early detection tests are double-edged swords that can save lives for some but bring unnecessary diagnoses for others.^20,21,22,23,24^ Along with cultural beliefs and commercial interests, media reporting has been regarded as one of the many drivers of overdiagnosis.^25^ A previous study investigating media coverage of early detection tests in stories across newspapers, magazines, and broadcast outlets found that most stories mentioned the benefits of these tests, whereas only one-third reported on their potential harms and even fewer mentioned the risk of overdiagnosis.^26^ Unlike traditional media, social media now allows anyone to create content and has a far greater reach. Furthermore, social media content is more relatable in the way it is delivered.^27^ Because it may have a greater influence on health behaviors than traditional media, social media needs to be explored further, including in relation to medical screening and diagnostic tests.

This study aimed to investigate how posts on leading social media platforms discuss popular medical tests, including full-body MRI, the multicancer early detection (MCED) test, and tests for antimullerian hormone (AMH), gut microbiome, and testosterone, which are targeted at generally healthy people and carry high potential for overdiagnosis or overuse. The specific objectives were to explore how often posts mentioned benefits and harms, including overdiagnosis or overuse; and the overall tone of the post. We also investigated whether posts from physicians, those with explicit evidence, and those with financial interests were more or less likely to be balanced in terms of the information on benefits and harms and in overall tone.

## Methods

### Study Design

We conducted a cross-sectional content analysis^28^ of information about medical tests in posts on the social media platforms Instagram and TikTok. A detailed protocol for the study was published previously.^29^ The data collected and analyzed in this study are public and largely reported in aggregate form. A waiver of consent from the University of Sydney Human Research Ethics Committee was obtained. The study reporting adheres to the Strengthening the Reporting of Observational Studies in Epidemiology (STROBE) reporting guideline.^30^

### Tests, Platforms, and Influencer Inclusion and Exclusion

Five tests were identified using the following criteria: (1) the existence of evidence or concerns that the test may not lead to improved health outcomes for generally healthy people, (2) the existence of evidence-based concerns about overdiagnosis or overuse, and (3) the tests being discussed on social media for use by generally healthy people. We also intended to identify a mix of tests targeted at individuals of varying sociodemographic characteristics, including sex and age, and including a range of costs.

The 5 tests identified were (1) full-body MRI, which claims to test for up to 500 conditions without evidence that any putative benefits outweigh the inevitable harms for healthy people and for which there are concerns about overdiagnosis^31^; (2) MCED tests, which are designed to detect circulating tumor–related mutated gene fragments in the blood and are being promoted to consumers as a way to screen for more than 50 cancers before symptoms arise, prior to full regulatory approval and absent clinical trial evidence that benefits will outweigh harms, including unnecessary diagnoses^32^; (3) the AMH test, also known as the egg-timer test, which is falsely promoted and sold as a fertility test,^33,34^ causing concerns about overuse and subsequent unnecessary fertility treatments; (4) the gut microbiome test, which takes a sample of stool and assesses the composition of microbiome in an individual’s gut, promising wellness via early detection of many conditions without good evidence of benefit and concerns about medical overuse causing harm and waste^35^; and (5) low testosterone (or low T) blood tests, which lack evidence of benefit for asymptomatic healthy men and are likely to cause overuse of supplements.^36^ The published protocol includes more detailed explanations about each test’s benefits and harms.^29^

Two of the most widely used and fastest-growing social media platforms across all age demographics were used for this study: Instagram and TikTok. Recent estimates suggest that Instagram has more than 2 billion active users per month, and TikTok has 1.5 billion.^37^ These platforms are predominantly for short-form content, such as infographics and reels (short videos), which has become more popular than longer-form content, such as long videos and blogs, in recent years.^38^

We included English-language social media posts from influential accounts, defined as more than 1000 followers and further categorized based on number of followers as nano (1000-10 000 followers), micro (10 000-100 000 followers), macro (100 000 to 1 million followers), and mega (≥1 million followers).^39^ Posts that discussed the previously mentioned tests from any individual (layperson or physician) or company account that were text-only posts, images, recorded videos, reels, and audio-only posts were included.

### Screening of Posts

Due to existing limitations in accessing data through platform-controlled application programming interfaces,^40^ this study used a manual approach to collect publicly available data. In January 2024, we created new Instagram and TikTok accounts and searched these platforms using the most common keywords related to each of the 5 tests, with keywords based on pilot testing (eMethods in Supplement 1). We searched and screened the top posts, as provided by the platform, against our inclusion and exclusion criteria until we had 100 eligible posts in each platform for each test (1000 in total). One screener (B.N.) screened posts, marking them as saved and sorting them into included or excluded folders (Instagram) or collections (TikTok). Another screener (J.R.Z.) independently verified the inclusion of eligible posts and exclusion of ineligible posts, with discrepancies resolved by discussion between the screeners. Of 2054 posts initially screened, 1054 were excluded because they did not discuss the test, they were not in English, or account holders had fewer than 1000 followers. Of the 1000 included posts, 18 were further excluded during data analysis as posts were no longer available or it was determined that they did not actually have an explicit mention of the test, leaving 982 posts in total. Posts were made between April 30, 2015, and January 23, 2024, with 1 post between 2015 and 2016, 7 posts between 2017 and 2018, 47 posts between 2019 and 2020, 313 posts between 2021 and 2022, and 614 posts between 2023 and 2024.

### Data Collection and Attributes

As described in the prospective protocol,^29^ data from the caption, audio and/or video, and on-screen text were coded into a developed and prespecified framework in Excel (Microsoft Corp) (eMethods in Supplement 1). To describe post and influencer or account-holder characteristics, we extracted publicly available data, including date of post; account name; number of followers; number of likes; number of comments; text, audio, and/or video content; length (if applicable); and whether the post was from an individual influencer (including physicians). We also extracted data on post attributes, including whether the posts mentioned information on benefits and harms (including overdiagnosis or overuse), the overall tone of the post (promotional vs neutral or negative), inclusion of explicit scientific evidence (eg, mentioning or showing reference to specific study findings), whether the post mentioned personal anecdotes, whether the post encouraged the viewer to take action and get the test, and whether clear financial interests were portrayed (eg, advertisement or selling of test, discount code, paid partnership or sponsorship noted, influencer discussion of receiving a free test).

### Statistical Analysis

The design of the coding spreadsheet was derived from similar previous research on media reporting of tests and treatments^26,41,42^ and was developed and refined iteratively by all authors. Two researchers (R.M. with B.N., E.G.G., T.C., or M.G.) independently used the sheet to code at least 20% of posts for each test (1 researcher per test) to evaluate the reliability of the spreadsheet. Level of agreement between the 2 coders across the attributes (eg, benefits and harms) was measured. Agreement was considered acceptable if κ >0.6 for a sample of at least 20% of posts. In some cases, level of agreement appears low using the κ statistic due to a high prevalence of a particular value for an attribute, known as the κ paradox.^43,44,45,46,47^ Therefore, we considered agreement acceptable if κ ≤0.6, but crude agreement was 85% or higher per our prospective protocol.^29^ In any cases where κ ≤0.6 and crude agreement was less than 85%, data were further independently double coded, using 20% blocks, until an acceptable level of agreement was met. Independent double coding of an initial 20% of posts reached acceptable agreement for almost all attributes across all tests. For 2 attributes across 2 of the tests, acceptable agreement was reached after a second 20% of posts were independently coded (eTable 1 in Supplement 1). Once agreement was acceptable, a researcher with experience in public health or overdiagnosis research coded the remaining posts (1 researcher per test).

We analyzed differences in attributes by test, platform, number of followers, and length using descriptive statistics (ie, numbers and percentages). The assumptions that physician posts, posts with evidence, and posts without clear financial interests were more likely to be balanced in terms of information on benefits, harms, and overall tone were statistically tested using univariate logistic regression models and reported using odds ratios (ORs) and 95% CIs. Analyses were unadjusted as per our preregistered protocol and due to a lack of theoretically plausible confounders in our dataset. The threshold for statistical significance was set at *P* < .05. Quantitative data were collated in Excel and analyzed in Stata SE, version 18.0 (StataCorp LLC). Exemplar quotations were collected to contextualize the main findings. Data on the themes of the posts will be reported elsewhere.

## Results

A total of 982 posts from accounts with a combined 194 200 000 followers (median [IQR] followers, 24 200 [4685-130 000] after excluding duplicate holders in our dataset) were analyzed. Of these 982 posts, 156 (15.9%) were from influencers identified to be physicians. Social media influencer and account holder characteristics by type of test are given in Table 1.

**Table 1.** Social Media Influencer and Account Holder Characteristics by Type of Test

Characteristic	No. (%) of posts^a^					
	All tests (N = 982)	MRI (n = 195)	MCED (n = 197)	AMH (n = 196)	Gut (n = 197)	Testosterone (n = 197)
Instagram	497 (50.6)	100 (51.3)	100 (50.8)	99 (50.5)	100 (50.8)	98 (49.7)
TikTok	485 (49.4)	95 (48.7)	97 (49.2)	97 (49.5)	97 (49.2)	99 (50.3)
No. of followers, median (IQR)^b^	24 200 (4685-130 000)	131 500 (16 500-382 000)	26 300 (3200-69 600)	10 900 (3607-47 100)	13 900 (3357-87 400)	30 800 (7887-118 600)
Total combined No. of followers^c^	194 200 000	101 100 000	55 344 742	6 767 457	9 390 826	21 630 368
Physician^d^	156 (15.9)	31 (15.9)	49 (24.9)	46 (23.5)	4 (2.0)	26 (13.2)

Abbreviations: AMH, antimullerian hormone; MCED, multicancer early detection; MRI, magnetic resonance imaging.

^a^ Data are presented as number (percentage) of posts unless otherwise indicated.

^b^ Followers of the account holders that made the posts.

^c^ The total combined followers of the included posts after excluding duplicate account holders. Note that followers could follow more than 1 account.

^d^ Influencers identifying as physicians.

**Figure 1.**
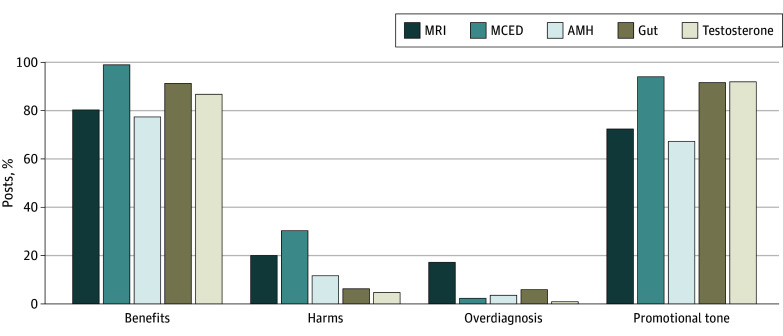
Social Media Posts on Medical Tests: Benefits, Harms, Overdiagnosis, and Tone AMH indicates antimullerian hormone; MCED, multicancer early detection; MRI, magnetic resonance imaging.

**Figure 2.**
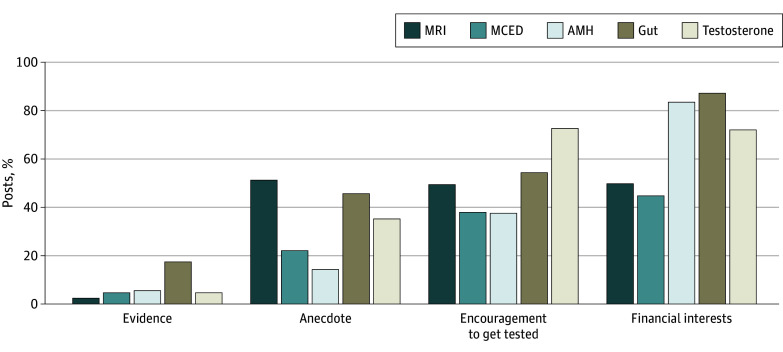
Social Media Posts on Medical Tests: Evidence, Anecdote, Encouragement, and Financial Interests AMH indicates antimullerian hormone; MCED, multicancer early detection; MRI, magnetic resonance imaging.

Benefits were mentioned in 855 posts (87.1%) and harms in 144 (14.7%), with 46 posts (4.7%) minimizing or diminishing any harms mentioned. The risk of overdiagnosis or overuse was only mentioned in 60 posts (6.1%). Overall, 823 posts (83.8%) had a promotional tone rather than neutral or negative (Table 2 and Figure 1). Evidence was explicitly used in 63 posts (6.4%), personal anecdotes were used in 333 posts (33.9%), 498 posts (50.7%) encouraged viewers to take action and get the test, and 668 account holders (68.0%) had financial interests (Table 2 and Figure 2). eTable 2 in Supplement 1 includes exemplar quotations for the attributes across the 5 tests.

**Table 2.** Social Media Post Attributes by Type of Test

Attribute	No. (%) of posts						
	All tests (N = 982)	MRI (n = 195)	MCED (n = 197)	AMH (n = 196)	Gut (n = 197)	Testosterone (n = 197)
Benefits	855 (87.1)	157 (80.5)	195 (99.0)	152 (77.6)	180 (91.4)	171 (86.8)
Harms	144 (14.7)	39 (20.0)	60 (30.5)	23 (11.7)	13 (6.6)	9 (4.6)
Harms minimized	46 (4.7)	4 (2.1)	39 (19.8)	0 (0.0)	0 (0.0)	3 (1.5)
Overdiagnosis	60 (6.1)	34 (17.4)	5 (2.5)	7 (3.6)	12 (6.1)	2 (1.0)
Promotional tone	823 (83.8)	142 (72.8)	186 (94.4)	132 (67.3)	181 (91.9)	182 (92.4)
Evidence	63 (6.4)	5 (2.6)	10 (5.1)	11 (5.6)	35 (17.8)	10 (5.1
Anecdote	333 (33.9)	100 (51.3)	44 (22.3)	28 (14.3)	91 (46.2)	70 (35.5)
Encouragement to get tested	498 (50.7)	97 (49.7)	75 (38.1)	74 (37.8)	108 (54.8)	144 (73.1)
Financial interests	668 (68.0)	98 (50.3)	89 (45.2)	165 (84.2)	173 (87.8)	143 (72.6)

Abbreviations: AMH, antimullerian hormone; MCED, multicancer early detection; MRI, magnetic resonance imaging.

eTables 3 to 5 in Supplement 1 include characteristics and attributes of posts by number of followers, platform, and length of posts. Posts from physicians were more likely to mention any potential harms of the tests (OR, 4.49; 95% CI, 2.85-7.06) and less likely to have an overall promotional tone (OR, 0.53; 95% CI, 0.35-0.80). Posts that included evidence were less likely to mention test benefits (OR, 0.49; 95% CI, 0.26-0.92), more likely to include potential harms (OR, 11.65; 95% CI, 6.77-20.08), and less likely to have an overall promotional tone (OR, 0.35; 95% CI, 0.20-0.62). Posts from account holders with clear financial interests were less likely to mention potential harms (OR, 0.24; 95% CI, 0.17-0.35), less likely to mention overdiagnosis (OR, 0.13; 95% CI, 0.06.-0.30), and more likely to have a promotional tone (OR, 6.34; 95% CI, 39.4-9.17). The rest of our analyses did not find statistically significant associations (Table 3).

**Table 3.** Logistic Regression Analyses Investigating the Influence of Being a Physician, Use of Evidence, and Financial Interest on Social Media Posts About Medical Tests

Attribute	Physician (n = 605)^a^		Evidence (n = 982)^b^			Financial interests (n = 982)^b^	
	Odds ratio (95% CI)	*P* value	Odds ratio (95% CI)	*P* value	Odds ratio (95% CI)		*P* value
Benefits	0.86 (0.53-1.39)	.54	0.49 (0.26-0.92)	.03	4.02 (2.73-5.91)		<.001
Harms	4.49 (2.85-7.06)	<.001	11.65 (6.77-20.08)	<.001	0.24 (0.17-0.35)		<.001
Harms minimized	1.29 (0.51-3.28)	.59	2.15 (1.00-4.64)	.05	0.85 (0.41-1.75)		.66
Overdiagnosis	1.10 (0.49-2.46)	.81	0.76 (0.35-1.63)	.48	0.13 (0.06-0.30)		<.001
Promotional tone	0.53 (0.35-0.80)	.003	0.35 (0.20-0.62)	<.001	6.34 (4.39-9.17)		<.001

^a^ n = 97 for analyses investigating harms minimized and overdiagnosis.

^b^ n = 144 for analyses investigating harms minimized and overdiagnosis.

## Discussion

Most of the almost 1000 evaluated posts about 5 popular medical tests from influential accounts on Instagram and TikTok were found to be misleading as they failed to mention potential harms. For all 5 tests examined, there are evidence-informed concerns about the risk of unnecessary diagnoses or treatments,^10,32,36,48,49^ yet only a small proportion of posts mentioned this potential harm. A few posts explicitly referred to evidence, whereas approximately one-third included personal anecdotes, and most account holders had some form of financial interest in promoting the test. Notably, posts from physicians were more likely to mention harms and less likely to have a promotional tone than other posts.

These results add to an increasing body of evidence, including from systematic reviews,^1,2^ finding misleading medical information is prevalent on social media. Although some posts demonstrated that the discussion of medical tests could indeed be more balanced, posts from social media platforms in this study overwhelmingly had a strong promotional tone, including the use of anecdotes as relatable and emotional appeals and encouragement to get tested. Such promotion has the potential to affect behavior, both negatively and positively, as previously suggested.^1,2^ Moreover, the lack of balance identified in this study is much greater than found in a previous analysis of media coverage^26^; therefore, combatting this problem will require different responses.^50,51^ The study results add weight to calls to develop and implement effective solutions to the problem of misleading medical information on social media.^12,13,52^ Chou et al^12^ have suggested development of “misinformation-related policies” within health care organizations, whereas Khullar^13^ has argued for the need to “‘prebunk’ medical myths” and “preemptively educate people about common misinformation techniques.” Given that physicians in this study were more likely to mention harms in their posts, they could play a stronger role in leading this social media reform. Clearly, this new evidence of persuasive and misleading information across almost 1000 posts, from accounts with combined followers of almost 200 million, demands responses to help curtail potential overdiagnosis and overuse.

In a historic move in May 2024, the US Food and Drug Administration introduced new regulations of medical tests, including those used to predict cancer risk, included in this study.^14^ Justification included concerns that alongside their benefits, tests can lead people to “initiate unnecessary treatment” and promotion can include “false and misleading claims.” Concurrently, and more broadly, there is increasing global concern about how to regulate and prevent misleading information on social media, with governments in many jurisdictions moving to action.^53^ The findings of this study offer rich new insights into the promotion of medical tests on social media and provide evidence that accurate and balanced social media posts exist and can be popular. These results provide important data for researchers, clinicians, consumers, and policymakers everywhere who are interested in understanding and preventing misleading and potentially harmful promotion of medical tests. Important next steps include more quantitative and qualitative investigation of social media promotion of tests, treatments, and diagnoses, including how these posts impact behavior, as well as identifying optimal strategies to respond to this problem.

### Strengths and Limitations

Strengths of this study include an innovative method adapting a previous media analysis^26,41^ for use on social media posts and a novel and timely investigation of tests currently being promoted to generally healthy people that carry risks related to overdiagnosis or overuse. This includes psychological harm of labeling, physical and environmental harm from unnecessary tests and treatments, and opportunity costs for health systems.^54^ The study also has some limitations. First, the sample for screening axiomatically relied on posts generated by platform algorithms, although a new account was created for screening and keywords were carefully identified through pilot testing. Second, we specifically did not analyze comments on posts, which may have provided additional insights. Third, as is common for studies of social media, it is impossible to verify the number of unique followers across all 5 tests. Fourth, we did not analyze specific categories of the attributes. For example, we did not look at whether the included evidence related to measuring test accuracy and predictive values or actual health outcomes, with the latter being far more meaningful in regard to benefits.

## Conclusions

In this cross-sectional study of social media posts on medical tests with potential for overdiagnosis or overuse, most posts were promotional, were from account holders with some form of financial interest in promoting the test, and mentioned test benefits. Only a few posts mentioned harms and/or provided reference to scientific evidence. These posts have the potential to mislead the public to getting tested despite the lack of evidence to support these tests and the potential for harms related to overdiagnosis or overuse. These findings indicate an urgent need for stronger regulation of misleading medical information on social media.

## Notes

Supplement 1. **eMethods 1.** List of Keywords Used to Search Tests **eMethods 2.** Coding Framework **eTable 1.** Kappas and Agreement Across All Tests **eTable 2.** Exemplar Quotes **eTable 3.** Characteristics and Attributes of Posts by Number of Followers **eTable 4.** Characteristics and Attributes of Posts by Platform **eTable 5.** Characteristics and Attributes of Posts by Video Duration (Tiktok Posts Only)

Supplement 2.
Data Sharing Statement
