# Supplementary material for: Social Media Posts About Medical Tests With Potential for Overdiagnosis
Source: JAMA Netw Open. 2025 Feb 26;8(2):e2461940. doi: 10.1001/jamanetworkopen.2024.61940 (PMC11866028; doi:10.1001/jamanetworkopen.2024.61940)
Supplement: Supplement 1. — eMethods 1. List of Keywords Used to Search Tests eMethods 2. Coding Framework eTable 1. Kappas and Agreement Across All Tests eTable 2. Exemplar Quotes eTable 3. Characteristics and Attributes of Posts by Number of Followers eTable 4. Characteristics and Attributes of Posts by Platform eTable 5. Characteristics and Attributes of Posts by Video Duration (Tiktok Posts Only) [file jamanetwopen-e2461940-s001.pdf]

Supplemental Online Content

Nickel B, Moynihan R, Gram EG, et al. Social media posts about medical tests with potential for overdiagnosis. *JAMA Netw Open*. Published online February 26, 2025. doi:10.1001/jamanetworkopen.2024.61940

eMethods 1. List of Keywords Used to Search Tests

eMethods 2. Coding Framework

eTable 1. Kappas and Agreement Across All Tests

eTable 2. Exemplar Quotes

eTable 3. Characteristics and Attributes of Posts by Number of Followers

eTable 4. Characteristics and Attributes of Posts by Platform

eTable 5. Characteristics and Attributes of Posts by Video Duration (Tiktok Posts Only)

This supplemental material has been provided by the authors to give readers additional information about their work.

© 2025 Nickel B et al. *JAMA Network Open*.

eMethods 1. List of keywords used to search tests*

	MRI	MCED	AMH	Gut	Testosterone
**Keyword 1**	Full-body MRI	Multi-cancer early detection test	AMH test	Microbiome test	Low testosterone test
**Keyword 2**	Whole-body MRI	Grail cancer test	AMH testing for female	Gut health test	Testosternone testing

*Platforms most common keywords based on piloting. Once saturation in terms of eligible posts were reached (i.e., there were no eligible posts immediately coming up) for keyword 1, keyword 2 were searched. On Instagram the second keyword was not needed for AMH and MCEDT.

© 2025 Nickel B et al. *JAMA Network Open*.

eMethods 2. Coding Framework

Item	*Description*	*Format*	*Additional coder notes (if applicable)*
**Unique ID**	Unique ID assigned to post	Pre-populated numeric ID	
**Platform**	Insta or TikTok (TT)	Pre-populated	
**Post URL**	URL to the post	Pre-populated	
**Date**	Date of Post	DD/MM/YYYY	Instagram has the month written in text. TikTok has the opposite date format. If no clear date for post – leave blank.
**Name**	Name of account	Free space for name	If two names appear for the account holder, try and discern which is the main one (usually the first or bolded one).
**Followers**	Number of followers	Free space for number	
**Eligible**	Is the post eligible?<br>'No' will be rare as posts have already been screened as eligible.	Drop Down with 2 options Yes/No	
**Likes**	Number of Likes for post (If not available =0)	Free space for number	
**Comments**	Number of Comments on post (If not available=0)	Free space for number	
**Test**	Name of test	Drop Down with 5 options: MRI/MCED/AMH/Gut/ Testosterone	
**Text**	Post includes text?	Drop Down 2options: Yes/No	
**Video**	Post includes a video?	Drop down with 2 options: Yes/No	Important to watch videos through to the end for important information.
**Length**	Length of video (if applicable). If video length is unknown, leave blank	Free space for Number in this format: 00:00	Leave blank for all Instagram posts as these posts do not specify their length.
**Audio**	Post includes Audio?	Drop Down with 2 options: Yes/No	
**Individual**	Is the account holder an individual?	Drop Down with 3 options: Yes/No/Unclear	Key/relevant information will be in the profile description/bio of the account holder. If it is a company account, code as No. Sometimes the account name sounds like a company, but there is a clear individual name underneath the company name, which can match the individual presenting the post – this would be a Yes. For posts by couples, code as No.

© 2025 Nickel B et al. *JAMA Network Open*.

**Physician**	Is account holder clearly a Physician? Only code this if coded Yes to the previous question.	Drop Down with 2 options: Yes or No/Unclear	Only code Yes to this if the individual is clearly a physician. You can usually find this information in the account holder profile description/bio. Sometimes this information will be in the post itself. The attire of the individual is irrelevant. A still image of a physician is irrelevant and does not mean the account holder is a physician. Simply describing themselves as a "Dr" or "Doctor" is similarly not sufficient, given they could be a PhD, or a doctor of homeopathy or nutrition who would therefore be coded as No.
**Benefits**	Potential benefits of the test implied or mentioned?	Drop Down with 3 options: Yes/No/Unclear	Any potential benefit of the test.
**Harms**	Potential harms or risks of the test implied or mentioned? If harms are mentioned but dismissed, code Yes.	Drop Down with 3 Options: Yes/No/Unclear	Any potential harm of the test. A caveat about whom the test may be applicable for, costs of the test, or the limits of what the test can do are not potential harms.
**Harms Minimised**	Are harms dismissed or played down? Only code this if you code Yes to Harms	Drop Down with 3 options: Yes/No/Unclear	Harms discussed but then dismissed or downplayed in some way to overemphasise the benefits.
**Overdiagnosis**	Is risk of overdiagnosis or overuse clearly implied or mentioned? Only code this if you code Yes to Harms	Drop Down with 3 options: Yes/No/Unclear	The words overdiagnosis or overuse do not have to be explicitly used.
**Evidence**	Is any scientific evidence clearly referenced to support claims about the test?	Drop Down with 3 options: Yes/No/Unclear	Clear or explicit reference to a piece of evidence, including an identifiable study, code as Yes. For example, showing an screenshot of the paper, mentioning the first author or conference abstract. Only stating “studies show” or “based on evidence” is not sufficient to be coded as Yes. Similarly, only mentioning the start of a trial, or a trial underway, without referring to trial results, does not constitute a Yes.
**Anecdote**	Is personal experience of the influencer themselves, or a personal anecdote, used, implied or mentioned in some way?	Drop Down with 3 options: Yes/No/Unclear	Personal experience of experience/anecdote concerning others, code as Yes.
**Encouragement to get the test**	Is there a direct encouragement (e.g., URL link provided) to book or buy a test? If any link to test sale, or discount code, in text or	Drop Down with 3 options: Yes/No/Unclear	Is there a specific link provided for viewer to take action and book or buy the test? If there is mention of "link in bio" - please click the link - and see if there is clearly any option to take action (e.g., book a test, book a consultation, buy a test, etc.). If there is,

© 2025 Nickel B et al. *JAMA Network Open*.

	primary comment or verbal or image.		code as Yes. There is no need to continue clicking on links - just the initial one. Simply having a hashtag is not a link, unless the viewer is being encouraged to use that hashtag to get tested. A link to get tested can include recommendations such as "DM me...." or highlighting a link on a mobile phone screen inside a video.
**Financial interests**	Is there any clear disclosure of any remuneration to influencer/account holder?	Drop Down with 2 Options: Yes or No/Unclear	Is there any clear financial interests that pertains to any financial or other gain the influencer or account holder may accrue by promotion of the test. If no clear disclosure, code as No. If the post is clearly an advertisement or there is mention of ad in the caption, audio/video or hashtag, code as Yes. If it is clear a free test was received by the account holder, code as Yes. If the account holder is promoting tests which are being sold, code as Yes. If the account is a company which is clearly selling the test, code as Yes. If the post mentions a discount code or paid partnership, collaboration or sponsorship in the caption, audio/video or in the list of hashtags, code as Yes. If it appears an individual or company could be selling the test (or offering consultations involving the test) and/or treatment/supplementation (e.g., testosterone), code as Yes.
**Overall Tone**	What is the overall tone of the post?	Drop Down with 3 options: Promotional / Negative / Neutral or Unclear	If there is a perceived intention to directly or indirectly promote the test, or if overall tone is positive, code as Promotional. If recommending against test, code as Negative. If the post covers benefits as well as harms and discussion around harms appears to outweigh benefits, code as Negative. If there is information on both the benefits and harms or the limitations of the test, code as Neutral.

© 2025 Nickel B et al. *JAMA Network Open*.

eTable 1. Kappas and agreement across all tests*

Attributes	Agreement Rate	Kappa
**MRI**		
Benefits	93.2%	0.69
Harms	97.7%	0.88
Overall tone	93.2%	0.78
Physician	86.4%	0.66
Evidence	97.7%	0.66
Anecdote	90.9%	0.74
Encouragment to get tested	88.6%	0.77
Financial interest	97.7%	0.95
**MCED**		
Benefits	92.5%	0.00
Harms	87.5%	0.66
Overall tone	90.0%	0.28
Physician	90.0%	0.84
Evidence	92.5%	0.78
Anecdote	87.5%	0.68
Encouragement to get tested	92.5%	0.82
Financial interest	82.5%	0.64
**AMH**		
Benefits	85.4%	0.63
Harms	92.7%	0.54
Overall tone	85.4%	0.99
Physician	97.6%	0.96
Evidence	100%	1
Anecdote	92.7%	0.78
Encouragement to get tested	82.9%	0.66
Financial interest	87.8%	0.63
**Gut**		
Benefits	100%	1

© 2025 Nickel B et al. *JAMA Network Open*.

Harms	100%	1
Overall tone	100%	1
Physician	95.0%	0.89
Evidence	100%	1
Anecdote	77.5%	0.56
Anecdote (2nd 40)	94.5%	0.89
Encouragement to get tested	65.0%	0.08
Encouragemenet to get tested (2nd 40)	89.9%	0.71
Financial interest	97.5%	0.00
**Testosterone**		
Benefits	87.2%	0.23
Harms	92.3%	0.36
Overall tone	79.5%	0.00
Overall tone (2nd 40)	94.9%	0.72
Physician	82.1%	0.71
Evidence	94.9%	0.47
Anecdote	97.4%	0.95
Encouragement to get tested	74.4%	0.48
Encouragement to get tested (2nd 40)	89.7%	0.69
Financial interest	87.2%	0.73

*20% of posts for each test (n=40) double coded. Due to the kappa paradox,[1-3] acceptable agreement if k was ≤0.6 but crude agreement was ≥85%, otherwise data was further independently double coded, using 20% blocks, (n=40) until acceptable level of agreement was met.

1. Feinstein AR, Cicchetti DV. High agreement but low kappa: I. The problems of two paradoxes. J Clin Epi. 1990;43(6):543-9.
2. Cicchetti DV, Feinstein AR. High agreement but low kappa: II. Resolving the paradoxes. J Clin Epi. 1990;43(6):551-8.
3. Zec S, Soriani N, Comoretto R, Baldi I. High Agreement and High Prevalence: The Paradox of Cohen's Kappa. Open Nurs J. 2017;11:211-218.

© 2025 Nickel B et al. *JAMA Network Open*.

eTable 2. Exemplar quotes

Attributes	Test	Exemplar quotes	Platform, year, followers
Benefits	MCED	"Scientists have discovered the holy grail of cancer detection."	TikTok, 2023, 14 100
Harms	MRI	"When getting routine MRI's, it's very common to see something abnormal, but are most often benign."	Instagram, physician, 2023, 1 million
Overdiagnosis	MCED	"We will likely find a lot of cancers earlier, but we still don't probably know what to do with them."	TikTok, 2023, 66 000
Promotional Tone	Gut	"You DESERVE to be healthy. It is your BIRTHRIGHT. These tests will let us know with CERTAINTY what is causing your specific health concerns."	Instagram, 2023, 68 500
Evidence	AMH	"According to the research, your AMH levels are actually NOT a good measure of 'ovarian reserve' NOR do they seem to impact chances of conception at all! In a recent large study of 750 women, no significant difference in fertility outcomes were seen between high and low AMH results PMID: 2656811."	Instagram, 2022, 2000
Anecdotes	Testosterone	"I took a blood test in my early 20s and found out I had the testosterone of a 70 year old man."	TikTok, 2023, 237 000
Encouragement to get tested	Testosterone	"If you're battling the motivation blues, consider checking those testosterone levels… reclaiming the mental mojo you need to conquer your ambitions."	Instagram, 2023, physician, 250 000
Financial interest	MRI	"The more we can create a demand for this kind of care…the faster prices will drop … That is the beauty of democratizing healthcare. I am forever grateful to the Prenuvo team for the life changing experience. If you are interested in learning more, visit their website and if you would like to book a scan yourself you can use the link … for $300 off."	Instagram 2023, 4 million

© 2025 Nickel B et al. *JAMA Network Open*.

eTable 3. Characteristics and attributes of posts by number of followers

Characteristics	<10,000 (n=346)	10,000 - <100,000 (n=353)	100,000 - <1,000,000 (n=238)	1,000,000+ (n=45)
Instagram, n (%)	246 (71.1%)	144 (40.8%)	87 (36.6%)	20 (44.4%)
TikTok, n (%)	100 (28.9%)	209 (59.2%)	151 (63.4%)	25 (55.6%)
Likes, mean (SD)	22 (8 to 59)	128 (47 to 459)	618 (229 to 2,378)	14,856 (1,562 to 39,374)
Physician, n (%)	36 (10.4%)	64 (18.1%)	47 (19.7%)	9 (20.0%)
Attributes				
Benefits, n (%)	303 (87.6%)	306 (86.7%)	204 (85.7%)	42 (93.3%)
Harms, n (%)	44 (12.7%)	50 (14.2%)	40 (16.8%)	20 (44.4%)
Harms Minimised, n (%)	14 (4.0%)	16 (4.5%)	12 (5.0%)	4 (8.9%)
Overdiagnosis, n (%)	8 (2.3%)	19 (5.4%)	26 (10.9%)	7 (15.6%)
Promotional tone, n (%)	306 (88.4%)	293 (83.0%)	188 (79.0%)	36 (80.0%)
Evidence, n (%)	20 (5.8%)	18 (5.1%)	17 (7.1%)	8 (17.8%)
Anecdote, n (%)	89 (25.7%)	117 (33.1%)	103 (43.3%)	24 (53.3%)
Encouragement to get tested, n (%)	190 (54.9%)	160 (45.3%)	128 (53.8%)	20 (44.4%)
Financial interests, n (%)	261 (75.4%)	235 (66.6%)	152 (63.9%)	20 (44.4%)

© 2025 Nickel B et al. *JAMA Network Open*.

eTable 4. Characteristics and attributes of posts by platform

Characteristics	Instagram (n=497)	Tik Tok (n=485)
Likes, mean (SD)	37 (11 to 242)	250 (71 to 1,260)
Physician, n (%)	53 (10.7%)	103 (21.2%)
Attributes		
Benefits, n (%)	449 (90.3%)	406 (83.7%)
Harms, n (%)	60 (12.1%)	84 (17.3%)
Harms Minimised, n (%)	24 (4.8%)	22 (4.5%)
Overdiagnosis, n (%)	17 (3.4%)	43 (8.9%)
Promotional tone, n (%)	449 (90.3%)	374 (77.1%)
Evidence, n (%)	31 (6.2%)	32 (6.6%)
Anecdote, n (%)	121 (24.3%)	212 (43.7%)
Encouragement to get tested, n (%)	335 (67.4%)	163 (33.6%)
Financial Disclosures, n (%)	398 (80.1%)	270 (55.7%)

eTable 5. Characteristics and attributes of posts by video duration (TikTok posts only)

Post characteristics	Under 1 minute (n=250)	1 minute + (n=231)
Likes, mean (SD)	226 (53 to 976)	298 (96 to 1,529)
Physician, n (%)	51 (20.4%)	51 (22.1%)
Attributes		
Benefits, n (%)	206 (82.4%)	198 (85.7%)
Harms, n (%)	26 (10.4%)	57 (24.7%)
Harms Minimised, n (%)	7 (2.8%)	15 (6.5%)
Overdiagnosis, n (%)	11 (4.4%)	31 (13.4%)
Promotional tone, n (%)	194 (77.6%)	178 (77.1%)
Evidence, n (%)	11 (4.4%)	20 (8.7%)
Anecdote, n (%)	95 (38.0%)	117 (50.6%)
Encouragement to get tested, n (%)	82 (32.8%)	78 (33.8%)
Financial Disclosures, n (%)	149 (59.6%)	118 (51.1%)

© 2025 Nickel B et al. *JAMA Network Open*.
