# Supplementary material for: Social Media Posts About Medical Tests With Potential for Overdiagnosis
Source: JAMA Netw Open. 2025 Feb 26;8(2):e2461940. doi: 10.1001/jamanetworkopen.2024.61940 (PMC11866028; doi:10.1001/jamanetworkopen.2024.61940)
Supplement: Supplement 2. — Data Sharing Statement [file jamanetwopen-e2461940-s002.pdf]

Data Sharing Statement

Nickel. Social Media Posts About Medical Tests With Potential for Overdiagnosis. *JAMA Netw Open*. Published February 26, 2025. doi:10.1001/jamanetworkopen.2024.61940

Data
Data available: Yes
Data types: Other (please specify)
Additional Information: Data sets
How to access data: The data sets generated and analyzed during this study are available from the corresponding author on reasonable request.
When available: With publication

Supporting Documents
Document types: None

Additional Information
Who can access the data: Corresponding author: Dr Brooke Nickel
brooke.nickel@sydney.edu.au
Types of analyses: Descriptive statistics and regression models.
Mechanisms of data availability: Upon reasonable request to the corresponding author.
Any additional restrictions: N/A
